# Mechanism of Fe(II) Chemisorption on Hematite(001) Revealed by Reactive Neural Network Potential Molecular Dynamics

**DOI:** 10.1021/acs.jpclett.4c03252

**Published:** 2025-01-16

**Authors:** Kit Joll, Philipp Schienbein, Kevin M. Rosso, Jochen Blumberger

**Affiliations:** † Department of Physics and Astronomy and Thomas Young Centre, University College London, London WC1E 6BT, United Kingdom; ‡ Lehrstuhl für Theoretische Chemie II, Ruhr-Universität Bochum, 44780 Bochum, Germany; ¶ Research Center Chemical Sciences and Sustainability, Research Alliance Ruhr, 44780 Bochum, Germany; § Pacific Northwest National Laboratory, Richland, Washington 99354, United States

## Abstract

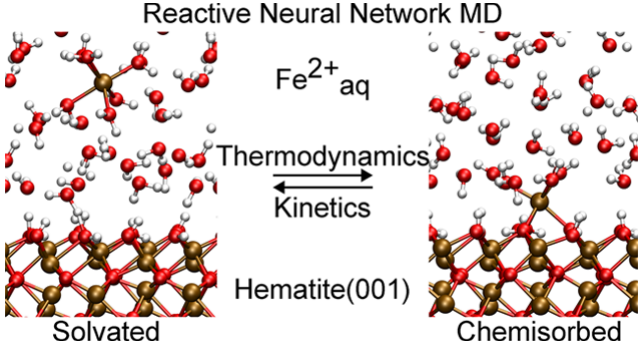

Atomic-scale understanding of important geochemical processes including sorption, dissolution, nucleation, and crystal growth is difficult to obtain from experimental measurements alone and would benefit from strong continuous progress in molecular simulation. To this end, we present a reactive neural network potential-based molecular dynamics approach to simulate the interaction of aqueous ions on mineral surfaces in contact with liquid water, taking Fe(II) on hematite(001) as a model system. We show that a single neural network potential predicts rate constants for water exchange for aqueous Fe(II) and for the exergonic chemisorption of aqueous Fe(II) on hematite(001) in good agreement with experimental observations. The neural network potential developed herein allows one to converge free energy profiles and transmission coefficients at density functional theory-level accuracy outperforming state-of-the-art classical force field potentials. This suggests that machine learning potential molecular dynamics should become the method of choice for atomistic studies of geochemical processes.

The interaction of mineral surfaces with aqueous ionic solutions plays an important role in many geochemical and biogeochemical processes including sorption, dissolution, nucleation and crystal growth.^1^ In these processes, solvated ions that are exchanged between bulk solution and the mineral surface, undergoing either solvation or desolvation reactions, get incorporated into or released from the crystal lattice and may induce a sequence of chemical (redox-) reactions. Experimental research probing mechanistic aspects of these elementary events is very challenging, particularly when the exchanging ions are chemically similar or when the ionic solutions are very dilute. In the case of identical ions differing only in their valence state, these challenges can be partially overcome using isotopic labeling techniques.^2−5^

Computational chemistry has been very valuable in complementing experimental studies, but the structural complexity of mineral/water interfaces, their chemical reactivity and the long time scales on which reactive events occur pose challenges for traditional simulation techniques. Previous classical molecular dynamics (MD) studies have revealed significant insights into adsorption of ions on mineral surfaces, yet remain limited by the resolution of physical and chemical interactions at the atomic scale due to the use of classical force fields.^6−8^ Density functional theory-based molecular dynamics (DFT-MD) has allowed us to study ion adsorption coupled to chemical bond breaking and formation from rigorous statistical mechanical principles, but the computational expense for large interfacial systems means that sampling of configurations is still rather limited.^9−12^ Here, machine learning (ML) molecular dynamics has emerged as a highly transformative approach that accurately predicts DFT-quality forces from a small set of training configurations, thereby boosting the accessible time scale of DFT-MD quality simulations by 3–4 orders of magnitude.^13−23^ These ML potentials, unlike the majority of their classical counterparts, are able to describe chemical reactions, bond breaking and formation akin to a reactive force field.^24^

Herein we devise a general approach for the development of an accurate committee high-dimensional neural network potential (c-NNP) for the simulation of ion adsorption on mineral oxide surfaces, using aqueous Fe(II) on α–Fe_2_O_3_ (hematite) as a model system.^15,25^ The adsorption of Fe(II) on hematite is the first step in a reaction sequence that contributes to the geochemical redox cycling of Fe species.^26−33^ Upon adsorption, Fe(II) can be oxidized to Fe(III) and the electron released moves across the oxide particle (via polaronic hopping^34^) to induce reduction reactions at remote surface sites (e.g., reduction of Fe(III) followed by release of Fe(II)).^33^ At neutral pH, the hematite (001) surface in contact with liquid water is largely terminated by hydroxyl groups and is net charge neutral, whereas Fe(II) in water forms a stable octahedral hexaquo-complex. For Fe(II) to adsorb on the surface, undergo electron transfer, and then be incorporated into the crystal lattice of hematite, the terminating hydroxyl groups of hematite need to replace the ion’s water ligands. Several mechanistic aspects of this process are unclear. Does this ligand exchange happen in a dissociative or associative fashion? Is this process exergonic and how fast is it compared to, e.g., the water ligand exchange reaction of aqueous Fe(II) in bulk solution? Is the reaction coupled to proton transfers or electron transfer to the oxide or both? Here we demonstrate that such questions can be investigated by exhaustive sampling of configurations at DFT-accuracy level using chemically reactive committee high-dimensional neural network potential molecular dynamics (c-NNP MD) simulations.

In this work we train a committee^25,35^ of second-generation Behler-Parrinello Neural Network Potentials^15^ (c-NNPs) to predict the potential energy of a given molecular system as a sum of atomic contributions using local atomic descriptors. Therein, atomic symmetry functions^36^ are employed within a spherical cutoff of about 6 Å, which has been proven suitable for aqueous systems.^16,17,21^ Like most other ML methods, our ML potential is therefore short-ranged and neglects explicit long-range electrostatics. For our given system, however, this is appropriate because (i) we only have one ion in the aqueous phases whose long-range effects are effectively screened by the surrounding water and (ii) we find that the force RMSE does not deteriorate for ion-surface distances exceeding the cutoff distance (Figure S3). See the Supporting Information (SI) for a more detailed discussion on the use of local descriptors.

Our approach is schematically illustrated in Figure 1. At first, we train two separate c-NNPs against DFT reference data (HSE06 with fraction of exact exchange adjusted to 12%),^16,34^ one for a model of the hematite (001)/liquid water interface and another for the Fe(II)-hexaquo ion in liquid water. The training data for the two systems are then merged to generate a single c-NNP for hematite (001)/liquid water including a Fe(II)-hexaquo ion in the water phase. Fe(II) is then slowly forced toward the surface by employing a series of umbrella potentials. Structures generated during the MD simulations that are unknown to the c-NNP (e.g., ligand exchange reactions or deprotonation reactions) are detected by spikes in disagreement of the potential energy predicted by the committee members. Such an event triggers a DFT reference calculation for these configurations in question followed by retraining of the c-NNP and resimulation of MD using the updated c-NNP. In this way the network iteratively learns all relevant configurations for physi- and chemisorption of aqueous Fe(II) on hematite(001) on the fly. Full simulation details are given in the SI.

**Figure 1:**
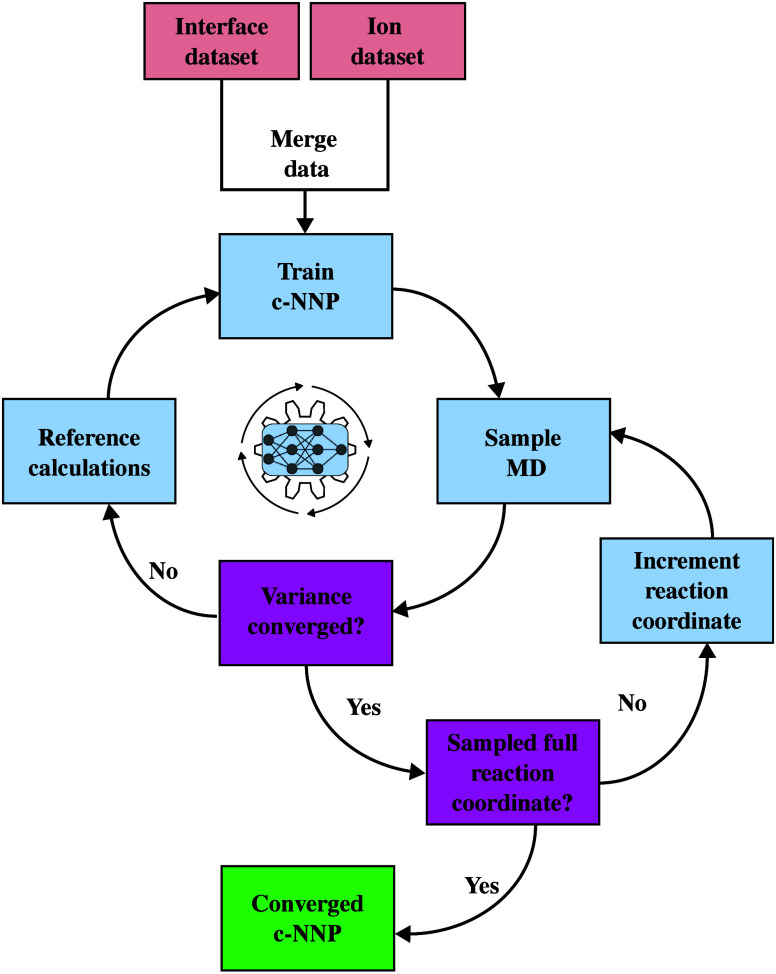
Scheme for generation of a c-NNP for adsorption of ions on solvated surfaces. First, two separate c-NNP models are trained, one for the solvated ion and another for the solvated surface (not indicated in scheme). This results in the ion and interface data sets (boxes in red) which are merged and used to train a new c-NNP model (first cycle, center). Using this model, umbrella sampling along a suitable reaction coordinate is used (here, distance to the surface) to force the ion toward the surface. Unknown configurations along the adsorption process are learned in the second cycle, bottom right. If the generated trajectory has a stable committee variance and the randomly extracted test configurations have a suitably low force error, the reaction coordinate is further incremented and the next umbrella window is sampled. Otherwise, the highest variance structures are extracted, reference calculations are performed, the data set is increased, and a new c-NNP model is trained. This protocol is iterated until the ion adsorption is complete and the c-NNP has a suitably low force error across the entire range of values for the reaction coordinate (box in green).

In the following we investigate the performance of the merged c-NNP on the two subsystems separately, hematite(001)/liquid water and Fe(II) in liquid water, before presenting results for Fe(II) adsorption on hematite(001). With regard to hematite(001)/liquid water, the merged c-NNP developed herein shows a performance (force root-mean-square-error (RMSE) = 131.1 meV Å^–1^) that is very similar to that of the c-NNP generated in our previous work that was trained on hematite(001)/liquid water only, i.e., without aqueous Fe(II) data, RMSE = 149.8 meV Å^–1^ .^16^ This means that the additional capability of the current c-NNP to describe both hematite(001)/liquid water and aqueous Fe(II) is not detrimental but even slightly improves the description of hematite(001)/liquid water. We note that the above RMSE values are somewhat higher when compared to recent literature RMSE values for simpler systems such as liquid water^37^ (≈40 meV Å ^–1^). The larger RMSE is due to the atoms of the hematite phase whose forces are more challenging to learn than those for the water phase, the latter having an RMSE of 59.0 meV Å^–1^ in line with recent literature values. This is likely related to the more complicated antiferromagnetic electronic structure of hematite. Despite that, the hematite equilibrium structure, bond length fluctuations and dynamics of terminating hydroxyl groups at the interface with liquid water is in very good agreement with DFT-MD, as shown in our previous work.^16^

The performance on aqueous Fe(II) is investigated by comparison to DFT-MD and experimental data. We find that the Fe–O radial distribution functions (Figure 2(A)) as well as the tilt angle distribution of first shell water molecules (Figure 2(B)) are in excellent agreement with the results from DFT-MD. The near quantitative agreement provides reassurance of the accuracy of the merged c-NNP to reproduce the DFT reference data. Neutron diffraction studies of the solvation structure of aqueous Fe(II) estimate the mean tilt angle of the first shell water molecules to be 32° with a standard deviation of 15°.^38,39^ The c-NNP yields a mean tilt angle of 33° with a standard deviation of 17°, DFT-MD simulations yield a mean tilt angle of 37° with a standard deviation of 17°. By contrast, the TIP3P-FB Fe(II)aq force field underestimates both the mean tilt angle (14°) and the standard deviation (8°) compared to experiment and DFT-MD simulations.^40^ This implies that the c-NNP is able to capture the finer structural details of the solvation structure of aqueous Fe(II) that are difficult to reproduce with a classical force field. Note that the DFT-MD simulations are limited by the high computational cost of hybrid functional DFT calculations, which limits the number of samples that can be generated.

**Figure 2:**
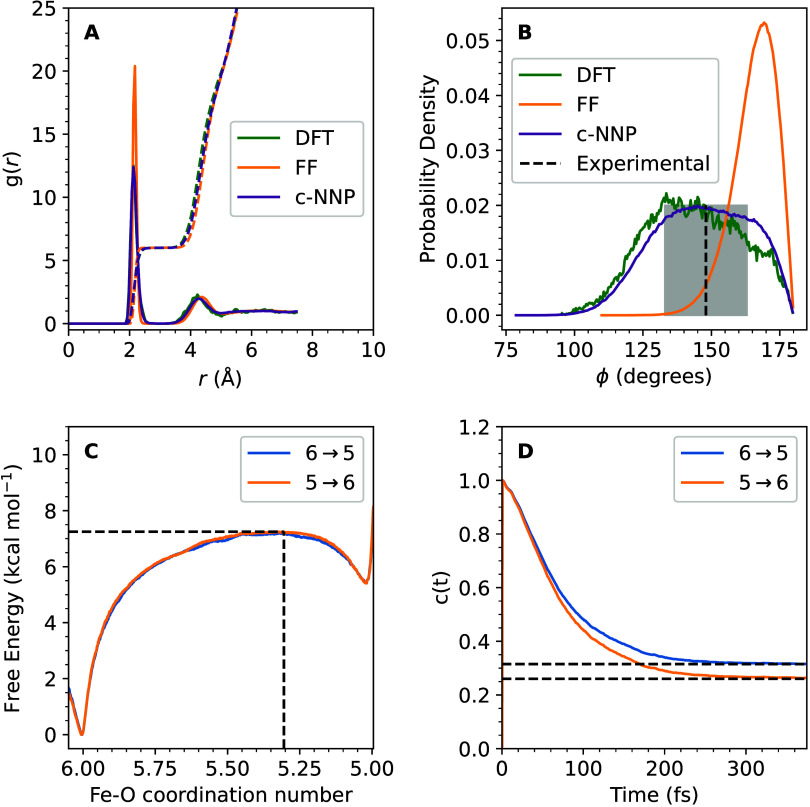
Structure and ligand exchange for Fe(II) in liquid water. The Fe–O radial distribution functions^41,42^ (RDFs) (A) and the tilt angle distribution of first shell water molecule (B) are shown for c-NNP MD (purple), DFT-MD (green) at an effective temperature of 300 K and the classical MD using the TIP3P-FB Fe(II)aq force field (orange) at 298 K. Note that for c-NNP MD and DFT-MD the resulting RDFs are hard to distinguish due to their almost quantitative agreement. The tilt angle is defined as the angle formed between the bisector of the two O–H bonds and the Fe–O vector. Experimental values for the tilt angle mean value and root-mean-square fluctuations are shown in dashed black lines and as a shaded gray bar, respectively. The free energy profiles eq 1 obtained from umbrella sampling in the CN = 6 → 5 direction (blue) and in the CN = 5 → 6 direction (orange) are shown in panel C, where the reaction coordinate *q* was taken to be the Fe(II) solvent coordination number CN, defined in eq S1. The normalized reactive flux correlation function, given by eq 9, is shown in panel D. The plateau value (dashed lines) is identified as the transmission coefficient, κ, according to eq 8.

Next, we investigate the performance of the c-NNP in describing the free energy barrier and rates for water ligand exchange, (Fe(II)(H_2_O)_5_H_2_O*)_aq_ + (H_2_O)_aq_ → (Fe(II)(H_2_O)_5_H_2_O)_aq_ + (H_2_O*)_aq_, where the water molecule leaving the first shell is annotated with an asterisk (*). Ligand exchange reactions may occur in a dissociative, interchange or associative mechanism. In the foremost mechanism the Fe–H_2_O* bond breaks leaving Fe(II) transiently 5-fold coordinated, followed by the take-up of a solvent molecule to regenerate the hexaquo complex. In the interchange mechanism a first shell water molecule is expelled and another bound simultaneously - akin to an SN2 mechanism. Notably for an interchange reaction, no intermediate is detectable. In an associative mechanism take-up of an additional solvent molecule leads to temporary expansion of the first coordination shell followed by expulsion of the H_2_O* ligand to regenerate the hexaquo complex. To be able to distinguish between the three mechanisms we use the difference in the coordination numbers, ΔCN = CN_1_ – CN_2_, between the Fe ion and the oxygen atoms of the six water molecules that are initially in the first coordination shell, CN_1_, and the coordination number between the Fe ion and the oxygen atoms of all remaining solvent molecules, CN_2_, as the reaction coordinate. The coordination number function is defined in the SI and uses a radial cutoff of 3 Å. Therefore, the reactant state is described by ΔCN = 6 (CN_1_ = 6, CN_2_ = 0) and the product state by ΔCN = 4 (CN_1_ = 5, CN_2_ = 1). The system is continuously transformed along ΔCN, from the reactant state (ΔCN = 6) to the product state (ΔCN = 4), using umbrella potentials in conjunction with c-NNP MD. In this way we do not bias the system toward a particular mechanism, as ΔCN = 5 is permitted with associative (CN_1_ = 6, CN_2_ = 1), dissociative (CN_1_ = 5, CN_2_ = 0) or interchange (CN_1_ = 5.5, CN_2_ = 0.5) mechanisms. We find that at ΔCN of about 5, Fe(II) has lost a first shell ligand and forms a pentaquo ion, corresponding to a dissociative mechanism. Alternative configurations corresponding to an interchange or associative mechanism are not observed. This is in line with the small positive activation volume reported experimentally (3.8 cm^3^ mol^–1^)^43^ and with the results obtained in previous transition path sampling simulations.^44^

A dissociative mechanism means that the activation free energy for the ligand exchange reaction is dominated by the activation free energy for dissociation of a water ligand. Using the coordination number (CN, defined in eq S1) between Fe(II) and the oxygen atoms of all water molecules as a reaction coordinate (*q*), we sampled the free energy profile from coordination numbers 6 → 5 (forward direction) and from 5 → 6 (backward direction) using umbrella sampling with the c-NNP. The free energy profile is defined according to eq 1,
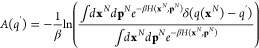
1where *H*(**x**^*N*^, **p**^*N*^) is the Hamiltonian, **x**^*N*^ and **p**^*N*^ the atomic coordinates and momenta, respectively, , where *T* is the temperature and *k*_B_ the Boltzmann constant, δ is the Dirac delta function and *q*′ is the value of the reaction coordinate at which the free energy is evaluated. The two profiles obtained from forward and backward sampling (Figure 2(C)) can be hardly distinguished from one another, indicating that the transformation has been reversibly sampled. We average the two profiles for calculation of thermodynamic and kinetic quantities (summarized in Table 1). We obtain a shallow minimum for the pentaquo complex, *ΔA* = 4.9 kcal mol^–1^ (eq 2) above the free energy of the hexaquo complex, and separated from it by an activation free energy of *ΔA*^‡^=5.1 kcal mol^–1^ (eq 4). Here the free energies were obtained by integration over the relevant parts of the free energy profile,
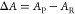
2
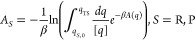
3
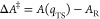
4where *ΔA* is the free energy difference, *A*_S_ is the free energy for reactant, *S* = R, and product, *S* = P, respectively, and *ΔA*^‡^ the activation free energy. R and P are characterized by minima on the free energy profile located at *q*_*S*,min_ and by stability regions defined by the interval [*q*_*S*,0_, *q*_TS_]. *q*_TS_ is the location of the transition state identified as the maximum of the free energy profile separating R and P and [*q*] is the unit of the reaction coordinate.

**Table 1:** Summary of Thermodynamic and Kinetic Quantities Calculated from the Free Energy Profiles of Water Exchange (Figure 2(C)) and Ion Adsorption (Figure 3)^a^

Transition			*ΔA*^b^	*ΔA*^TS^^c^	*ΔA*^‡^^d^	ν^e^	κ^f^	*k*^g^
5-fold	→	6-fold	–4.9	1.8	0.2	2.7 × 10^12^	0.26	5.1 × 10^11^
6-fold	→	5-fold	4.9	7.2	5.1	2.9 × 10^12^	0.32	1.8 × 10^8^
Non	→	Physi	–1.6	1.4	0.8	1.2 × 10^12^	0.20	6.5 × 10^10^
Physi	→	Non	1.6	3.0	2.3	1.2 × 10^12^	0.17	3.9 × 10^9^
Physi	→	Mono	–1.1	4.3	3.7	1.2 × 10^12^	0.08	2.0 × 10^8^
Mono	→	Physi	1.1	5.8	4.8	1.2 × 10^12^	0.05	2.0 × 10^7^
Mono	→	Tri	–7.1	2.5	1.5	1.2 × 10^12^	0.03	3.4 × 10^9^
Tri	→	Mono	7.1	9.4	8.5	1.2 × 10^12^	0.02	1.3 × 10^4^

a Free energy differences (*ΔA*), free energy barrier heights (*ΔA*^TS^), activation free energies (*ΔA*^‡^), frequency prefactors (ν), transmission coefficients (κ), and reactive flux reaction rate constants (*k*). All free energies are reported in units of kcal/mol. Frequency prefactors and rate constants are reported in units of s^–1^.

b Equation 2.

c Free energy barrier height, *ΔA*^TS^ = *A*(*q*_TS_) – *A*(*q*_R,min_), where *A*(*q*_TS_) and *A*(*q*_R,min_) are the free energies eq 1 at the transition state *q*_TS_ and at the reactant minimum *q*_R,min_, respectively.

d Equation 4.

e Equation 7. Note that ν may differ for forward and backward reactions for reaction coordinates that are not linear functions of atomic coordinates, such as *q* = CN (eq S1).

f Equation 8.

g Equation 5.

The reaction rate constants (*k*) for forward and backward reactions are obtained from the reactive flux formalism,^45,46^
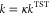
5where *k*^TST^ is the transition rate constant,
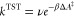
6Δ*A*^‡^ is the activation free energy defined in eq 4 and ν is the frequency prefactor,
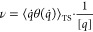
7with θ denoting the Heaviside function, *q̇* the time derivative of *q* at time *t* = 0 and ⟨···⟩_TS_ the canonical ensemble average at the transition state. κ is the transmission coefficient,
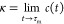
8defined by the plateau value of the normalized reactive flux correlation function *c*(*t*) that is reached after time τ_m_,
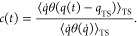
9For calculation of the flux correction function eq 9 we carry out 200 ps c-NNP MD simulations constrained to the transition state, from which we select 128 configurations in equidistant intervals. For each of these configurations, we generate 128 sets of initial velocities drawn from a Maxwell–Boltzmann distribution and propagate 128^2^ (16384) short trajectories using the c-NNP (Figure 2(D)). We also calculate the velocity of the reaction coordinate at *t* = 0 from these initial conditions via finite difference to obtain the frequency prefactor, ν, eq 7. For more details on these calculations we refer to the SI.

The normalized flux correction function is shown in Figure 2(D). We obtain a transmission coefficient of about 0.3 in the forward and reverse direction indicating that simple transition state rate theory would overestimate the reaction rate by a factor of about 3. We obtain a reactive flux rate estimate *k* = 1.8 × 10^8^ s^–1^ for CN = 6 → CN = 5 and 5.1 × 10^11^ s^–1^ for CN = 5 → CN = 6. The experimental value for the rate constant of water exchange for Fe(II) in water is 4.4 × 10^6^ s^–1^ at 298 K,^43^ which is approximately 40 times slower than our computed rate constant for CN = 6 → CN = 5 that limits the kinetics of the water exchange reaction. This discrepancy is likely due to the HSE06 density functional used to train the c-NNP. In order to yield the correct rate, assuming identical values for κ, the activation free energy would need to be *ΔA*^‡^ = 7.3 kcal mol^–1^, 2.2 kcal mol^–1^ higher than the value obtained from the c-NNP. It is well-known that the HSE06 functional underestimates reaction barriers due to the remaining electron self-interaction error, which we attribute to being the cause of our somewhat overestimated rate.^47^

Having benchmarked the c-NNP on the Fe(II) water-exchange reaction, we now proceed with the simulation of adsorption of aqueous Fe(II) on the hematite(001) surface. We carry out umbrella sampling to obtain the free energy profile for adsorption using the distance between the Fe(II) ion and the surface oxygen layer in the direction of the surface normal as the reaction coordinate. Using 14 umbrella windows covering a distance range between about 1 to 8 Å we find that the free energy profile converges only after about 400 ps per window (see Figure S7), which is well beyond the simulation time accessible to DFT-MD. The final free energy profile (500 ps simulation time per window; 7 ns in total) and representative snapshots corresponding to maxima and minima on the profile are shown in Figure 3. One can clearly identify 4 different stable complexes for Fe(II): nonadsorbed, physisorbed (outer-sphere complex), monodentate and tridentate chemisorbed (inner-sphere complexes). The Fe–O bond lengths for these structures are summarized in Tab. S4. The thermodynamic and kinetic quantities obtained from the free energy profile and from reactive flux sampling at the transition states connecting the stable structures are summarized in Table 1.

**Figure 3:**
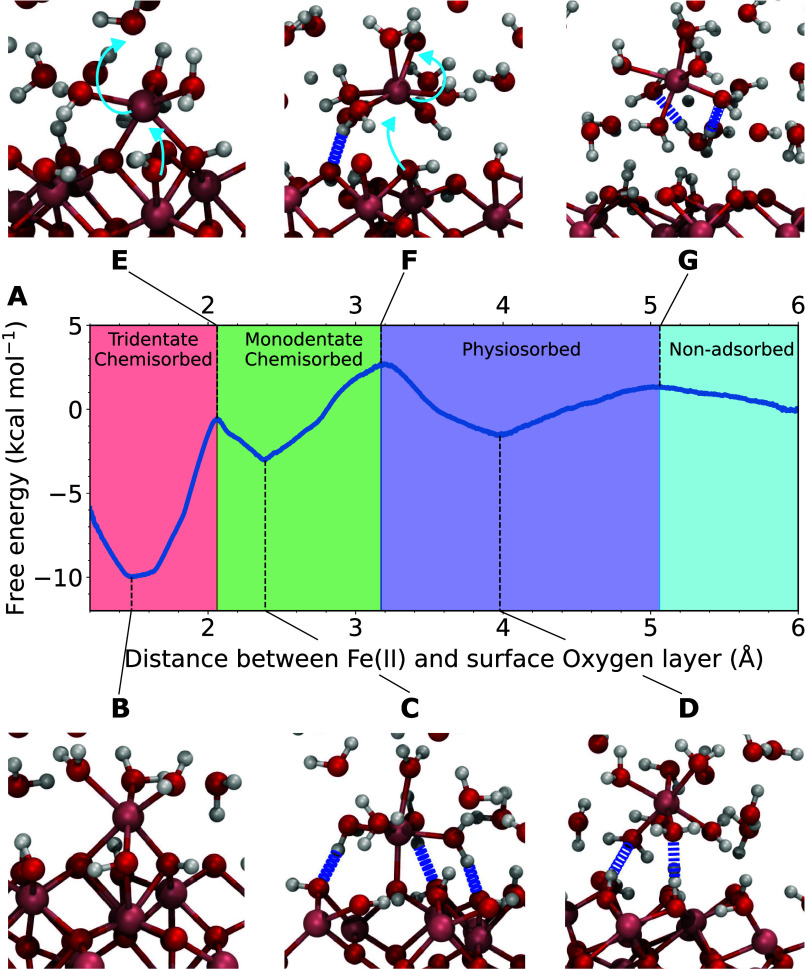
Free energy profile for adsorption of Fe(II) on hematite(001) in aqueous solution. In (A) the free energy profile eq 1 is shown as a function of the distance of the ion from the surface along the surface normal. The latter is obtained from the mean position of all oxygen atoms terminating the surface. The free energy was obtained from umbrella sampling using the c-NNP as outlined in the main text (see SI for details). The insets show representative snapshots along umbrella sampling trajectories for stable structures corresponding to local minima on the free energy profile: tridentate chemisorbed (B), monodentate chemisorbed (C) and physisorbed (D). In addition, representative transition state structures are shown corresponding to local free energy maxima: monodentate → tridentate chemisorbed (E), physisorbed → monodentate chemisorbed (F) and nonadsorbed → physisorbed (G). Iron atoms are shown in pink, oxygen atoms in red and hydrogen atoms in white. Selected hydrogen bonds are shown in blue. Thermodynamic and kinetic properties obtained from the free energy profile are summarized in Table 1.

The nonadsorbed Fe(II)-hexaquo complex has the same structure as the Fe(II)-hexaquo complex in bulk solution. It exists up to distances of about 6 Å from the hematite surface, at which point it is separated from the surface by the first solvation shell and an additional water layer. Decrease of the distance to about 5 Å squeezes the additional water layer out of the interfacial space (Figure 3(G)) resulting in the formation of a stable physisorbed Fe(II)-hexaquo complex at about 4 Å (Figure 3(D)). This process is associated with a small activation free energy of 0.8 kcal mol^–1^ and it is weakly exergonic by 1.6 kcal mol^–1^. The physisorbed complex is held to the surface mainly by two strong hydrogen bonds donated by two surface hydroxyl groups pointing in the direction of the surface normal and accepted by two adjacent first shell water molecules of Fe(II).

Further approach of Fe(II) toward the surface results in a partial rupture of a coordination bond between Fe and a first shell water molecule and a simultaneous decrease in the distance between Fe and a surface hydroxyl group (Figure 3(F)). Notably, the O–H bond of the latter points in a direction parallel to the surface, leaving the oxygen atom free to coordinate the incoming Fe atom. This process generates a stable monodentate chemisorbed ≡O(H)-Fe(II)-(H_2_O)_5_ species at about 2.4 Å that is stabilized by, on average, three hydrogen bonds between surface hydroxyls and first shell water molecules (Figure 3(C)). The process is weakly exergonic by 1.1 kcal mol^–1^ and the activation free energy is 3.7 kcal mol^–1^.

Finally, further approach of the Fe toward the surface triggers two rapid successive substitutions of two first shell water molecules by two surface hydroxyl groups that have their O–H bond pointing parallel to the surface (Figure 3(E)). This results in the formation of a very stable tripod-like tridentate chemisorbed (≡O(H))_3_-Fe(II)-(H_2_O)_3_ species at a distance of about 1.5 Å (Figure 3(B)) that is about 7.1 kcal mol^–1^ lower in free energy than the monodentate chemisorbed species and separated from it by a barrier of 1.5 kcal mol^–1^. The relatively high exergonicity for formation of the tridentate structure is mainly due to an increase in the bond strength of all Fe–O bonds, as indicated by a significant decrease of all six Fe–O bond distances by about 0.06 Å compared to the ones in the monodentate structure. The bidentate chemisorbed structure is found to exist only transiently. It converts very rapidly into the tridentate chemisorbed structure and does not correspond to a local minimum on the free energy profile.

We find that the oxidation state of Fe remains +2 along the adsorption process, from nonadsorbed to tridentate chemisorbed, i.e., we do not observe spontaneous electron transfer from Fe(II) to hematite as Fe(II) approaches the surface. This was concluded by considering the Hirshfeld spin moment on the adsorbing Fe(II) ion for 140 configurations sampled along the free energy profile (10 from each window). These configurations yielded a Hirshfeld spin moment of 3.66 ± 0.04 μ_*B*_ which is very close to the value obtained from DFT-MD simulations for aqueous Fe(II), 3.68 ± 0.02 μ_*B*_, and different from the value for aqueous Fe(III), 4.22 ± 0.01 μ_*B*_.

The surface hydroxyl groups forming the coordination bonds with Fe(II) do not spontaneously deprotonate on the nanosecond time scale of present simulations. Instead, we observe proton transfer (PT) events, primarily involving the water ligands of Fe(II). Typically, a first-shell water molecule deprotonates, forming e.g. monodentate ≡O(H)-Fe(II)-(OH)(H_2_O)_4_, with subsequent PT to surface hydroxyls via a water relay. While there are many of these types of species, they are predominantly short-lived (on the order of 10–250 fs). However, our findings indicate that some PT events, particularly near the monodentate to tridentate transition state, can persist significantly longer (e.g., up to 40 ps). These observations suggest that while transient PT events are common, longer-lived PT events do occur but are infrequent on the 7 ns time scale of our adsorption simulations.

It is likely that the rate constants for Fe(II) adsorption are overestimated by 1–2 orders of magnitude when considering our results for the water exchange reaction of aqueous Fe(II) where similar Fe–O bonds are broken and formed. Even if this is so, our simulations predict that Fe(II) adsorption is fairly rapid (at least on the order of microseconds), in qualitative agreement with the experimental observations.^48^ The relatively high exergonicity of the overall adsorption process means that escape of Fe(II) from the surface is fairly slow once the tridentate structure has formed. This will give tridentate chemisorbed Fe(II) sufficient time to transfer an electron to hematite and to thus become firmly incorporated as a Fe(III) ion into the crystal lattice.

Here it is pertinent to compare the free energy profiles obtained from classical FF MD^6^ and c-NNP MD, as there are marked similarities and differences. First, from the classical MD studies, the same 4 stable species are identifiable: nonadsorbed, physisorbed, monodentate chemisorbed and tridentate chemisorbed. The Fe-surface distances at which the corresponding free energy minima and maxima occur are also very similar (see SI for details). Yet, the adsorption profile obtained from classical force field MD is strongly endergonic, in contrast to the profile obtained from the c-NNP. Only after the removal of 2 H atoms from surface hydroxyl groups, was the adsorption process exergonic with the classical force field methodology. However, these surface conditions are unexpected because it is known experimentally that the surface remains charge-neutral over a pH range of 4–14.^49^ The previously predicted endergonicity of Fe(II) adsorption on the fully hydroxylated surface might be due to some deficiencies in the force field used. First, in the force field description the terminating O–H bonds have a preference to point along the surface normal (out-of-plane). In this configuration the surface oxygen atom cannot coordinate the incoming Fe(II) and energy input is required to reorient the hydroxyls to the reactive in-plane configuration, providing an additional barrier to adsorption. By contrast, in c-NNP and DFT-MD the hydroxyl groups readily interconvert between in-plane and out-of-plane orientations, giving an average ratio of about 1:1.^16,50^ This results in exposed surface hydroxyl oxygens ready for coordination with incoming Fe(II). Second, in the force field description of the tridentate structure, the Fe–O bonds with the surface hydroxyl groups (2.22–2.25 Å) are significantly longer and thus weaker than with the water molecules in the hexaquo complex (2.07–2.09 Å). This is in contrast to the c-NNP, where in the tridentate structure the Fe–O bonds with the hydroxyl oxygens are similar (2.16 Å) and with the remaining 3 water ligands even stronger (2.11 Å) than with the water molecules in the hexaquo complex (2.17 Å). These two major differences between classical force field and c-NNP likely explain the different energetics for Fe(II) adsorption.

In summary, this study successfully employs neural network potentials to achieve a high-resolution understanding of Fe(II) adsorption at the hematite (001)/liquid water interface, bridging computational innovation with new atomistic insight for elementary geochemical processes. The c-NNP methodology provides estimates of the kinetics and detailed free energy profiles for water-exchange reactions and ion adsorption processes with an accuracy that is limited by the accuracy of the DFT functional used to train the network. By revealing the structural intricacies of various adsorption complexes and their energetics, the study demonstrates the advantages of modern ML potentials over classical force fields. These findings not only enhance our understanding of Fe(II) adsorption mechanisms on geologically important minerals, but also pave the way for future research in environmental remediation and energy conversion applications, emphasizing the critical role of electronic structure and surface chemistry in geochemical processes. Future work will involve calculating electron transfer rates for each adsorbed complex and transition state identified in this study using constrained DFT.^34,51^ This will allow us to determine the overall electron transfer rate of oxidative adsorption, which will provide a more comprehensive understanding of the electron transfer dynamics at the mineral-water interface and the geochemical redox cycling of Fe species. Furthermore, we plan to study how the application of electric fields perpendicular to the surface affects the adsorption process, using the c-NNP method in conjunction with a finite field ML approach.^10,52^

## Supporting Information Available

Detailed descriptions of the computational methodologies, additional figures illustrating key results, complete tables of umbrella sampling potentials and force constants, error analysis for c-NNP training and testing, reactive flux analysis protocols, and convergence analysis data (PDF)
