# Supplementary material for: Mechanism of Fe(II) Chemisorption on Hematite(001) Revealed by Reactive Neural Network Potential Molecular Dynamics
Source: J Phys Chem Lett. 2025 Jan 16;16(4):848–56. doi: 10.1021/acs.jpclett.4c03252 (PMC11789133; doi:10.1021/acs.jpclett.4c03252)
Supplement: Supplementary file 1 — jz4c03252_si_001.pdf [file jz4c03252_si_001.pdf]

Supporting Information: Mechanism of Fe(II) chemisorption on hematite (001) revealed by reactive neural network potential molecular dynamics

Kit Joll,[†] Philipp Schienbein,[†,‡,¶] Kevin M. Rosso,[§] and Jochen Blumberger[*,†]

†*Department of Physics and Astronomy and Thomas Young Centre, University College London, London, WC1E 6BT, United Kingdom*

‡*Lehrstuhl für Theoretische Chemie II, Ruhr-Universität Bochum, 44780 Bochum, Germany*

¶*Research Center Chemical Sciences and Sustainability, Research Alliance Ruhr, 44780 Bochum, Germany*

§*Pacific Northwest National Laboratory, Richland, Washington 99354, United States*

E-mail: j.blumberger@ucl.ac.uk

Computational Details

DFT calculations

All three systems investigated, aqueous Fe(II), hematite(001)/liquid water and aqueous Fe(II) in contact with hematite(001), were treated at the same hybrid level of DFT, computed with `cp2k`, using the quickstep module.[1,2] Namely, a modified version of the HSE06 functional was used, where the fraction of exact exchange is reduced from 25% to 12%, supplemented with a D3 dispersion correction.[3–7] This functional has been shown to reproduce several important experimental properties of hematite such as the crystal structure, band gap, antiferromagnetic ordering and spin density distribution.[4,5] A TZVP-MOLOPT-GTH basis set was used for Hydrogen, while DZVP-MOLOPT-SR-GTH basis sets were employed for Iron and Oxygen.[8] Norm-conserving relativistic Goedecker-Teter-Hutter (GTH) pseudopotentials were employed to represent the core electrons. To efficiently include the exact exchange contribution in the hybrid calculation, the auxiliary density matrix method (ADMM) was utilised. To this end, the electron density for the exact exchange calculation was represented in a smaller auxiliary basis, with AUX FIT cpFIT3 for Oxygen and Hydrogen, and AUX FIT cpFIT11 for Iron.[9] A kinetic energy cutoff of 600 Ry was utilised for the plane wave basis set, with a relative cutoff of 40 Ry. For simulations of aqueous Fe(II), spin-unrestricted Kohn-Sham density functional theory was employed with a total charge of 2+ and a spin multiplicity of 5 (high spin), in accordance with the experimental spin ground state. Calculations of hematite(001)/liquid water were carried out as described in Ref.[7] The O-terminated (001) surface was fully hydroxylated and charge neutral, the Fe(III) atoms had a spin multiplicity of 6 (high spin) and the spins in adjacent Fe layers were antiferromagnetically aligned, in accordance with the experimental spin ground state. The same spin states were chosen to model aqueous Fe(II) in contact with hematite(001).

DFT MD with GGA or hybrid DFT functionals (including HSE06 with original or reduced fraction of exact exchange) as well as c-NNP MD trained on these functionals tend to

overstructure liquid water and underestimate self-diffusion.[10–12] For DFT MD, this shortcoming has commonly been cured by increasing the simulation temperature to counterbalance the overstructuring.[10,12–14] Likewise, in our previous study we have shown that DFT MD and c-NNP MD at a simulation temperature of 400 K yields both accurate RDFs and the self-diffusion coefficient for liquid water when compared to experimental data at 300 K.[7] Therefore, we have chosen a target temperature of 400 K in all DFT MD and c-NNP MD simulations reported in this work and refer to this temperature as an "effective temperature of 300 K" in the main text.

Training and testing of c-NNP for aqueous Fe(II)

To model the aqueous Fe(II) ion, a cubic simulation box of side length a = 15.52125 Å was used containing one Fe(II) ion and 125 water molecules, corresponding to a density of 1.025 g cm^{-3} and concentration of 0.444 M. To obtain the initial structure, the box side length was chosen to reproduce the density of water to be 1 g cm^{-3}, for 125 water molecules. Then the box was discretized into a 5x5x5 grid and a random rotation matrix was applied to each water molecule before being inserted at a given grid point. Finally, the Fe(II) ion was inserted at the center of the box. We used the recently reported force field parameters for Fe(II) in combination with TIP3P-FB water[15] to energy minimize and equilibrate the initial structure to 298 K (see below for further details of classical MD simulations). After a 20 ps equilibration of this initial geometry to 298 K, a 2 ns classical MD trajectory was simulated to generate structures for an initial c-NNP as detailed in the following.

An initial c-NNP model is trained on DFT energies and forces calculated on 10 configurations along the aforementioned classical MD trajectory which were sampled every 100 ps from the second half of the trajectory. The c-NNP model was set up in the following manner: 8 committee members were employed for the active learning procedure, each committee member used generic symmetry functions,[16] 2 hidden layers each containing 25 nodes and were optimised with the Kalman filter using the `n2p2` package.[16,17] Active learning was it-

erated against the aforementioned classical MD trajectory until a converged variance of the committee was obtained across the entire trajectory. This also corresponded to a force RMSE vs DFT that plateaued with respect to the number of structures in the training set.

When these two criteria were met, the c-NNP was used to run MD simulations to explore the configuration space. These simulations were performed at 400 K using the Nose-Hoover thermostat.[18,19] When the network crashed, the training set was augmented by sampling configurations prior to the crash and the active learning procedure was repeated. The variance of the committee members was monitored to ensure that only physically meaningful configurations were added to the training set. This procedure was repeated until the potential was stable for a nanosecond of c-NNP MD. The resultant potential is referred to as the final c-NNP for the Fe(II)aq system. Notice that in the second training step leading to the final c-NNP, the system is allowed to explore the equilibrium structures that would be produced in a DFT MD simulation. In the first step, only FF MD structures are considered, and what the DFT forces would be if those structures were produced. Upon simulating MD with a network trained on those FF MD configurations, the system relaxes into the DFT MD configurations, requiring iterative retraining until stability is reached.

To test the c-NNP force prediction, a 75 ps simulation was performed. The starting configuration was taken from a 50ps equilibration run that began with an equilibrated DFT MD configuration. Then 20 random configurations were extracted. These c-NNP MD simulations were run with a 0.5 fs timestep, in the `NVT` ensemble with the CSVR[20] thermostat. The force RMSE of the committee members was calculated, see Fig. S1. Overall, this procedure resulted in a training dataset containing 130 configurations with a total force RMSE of 60.4 meV $Å^{-1}$ on a test set of 20 configurations randomly sampled from the 75ps trajectory. Structural benchmarking was also performed by calculating the radial distribution function and tilt angle distribution of the water molecules around the Fe(II) ion. Unremarkably, the c-NNP MD simulations were in excellent agreement with the DFT reference data. This is to be expected because dynamic properties are a direct result of the forces, and the forces

were well predicted by the c-NNP.

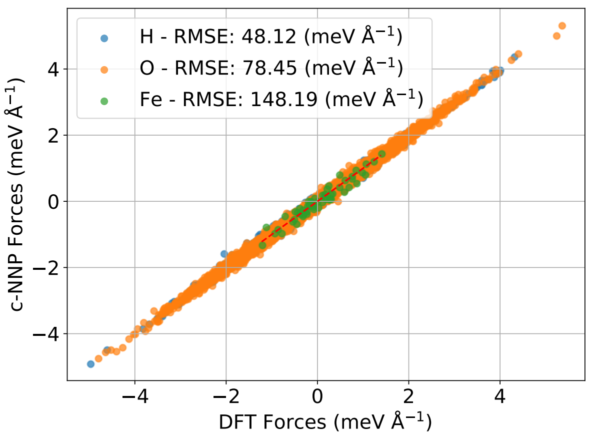

Figure S1: Test of c-NNP for aqueous Fe(II) against DFT reference data. Atomic forces were calculated for 20 configurations randomly sampled from a 75 ps c-NNP trajectory. See SI text for details.

Training and testing of c-NNP for hematite(001)/liquid water

For modelling the aqueous hematite interface, the same simulation box was utilised as outlined in the previous study.[5,7] Namely, a 435 atom simulation cell with 93 water molecules solvating an O-terminated (001) plane of a $2 \times 2 \times 1$ supercell of hematite. This yielded a simulation cell with lattice vectors of $a = 10.241$ Å, $b = 10.2943$ Å, $c = 47.3423$ Å and $\alpha = 91.966°$, $\beta = 87.424°$, $\gamma = 119.738°$, with a water density of 0.997 kg L^{-1}. Note that hydroxyl crystal termination results in a charge-neutral surface. These -OH groups remain protonated throughout nanoseconds of simulation due to their high pKa, which has been estimated to be 18.5.[21] Furthermore, previous investigations have shown the surface to be charge-neutral over a wide range of pH values.[22] This dataset was trained using the same DFT setup outlined in the DFT reference calculation section, and achieved a total force error of 149.8 meV Å^{-1}.[7] The parameters for this network are identical to that for aqueous Fe(II) above, with the appropriate symmetry functions included to describe hematite.

Training and testing of c-NNP for aqueous Fe(II) in contact with hematite(001)

The scheme for training the c-NNP for aqueous Fe(II) in contact with hematite(001) is shown in Fig. 1 in the main text. 398 structures of the hematite(001)/water interface and 130 structures of aqueous Fe(II) were merged to generate an initial c-NNP that describes both systems. Then an initial configuration of hematite(001)/liquid water with the water phase containing a Fe(II) ion was generated. To this end, an equilibrated configuration of the hematite(001)/liquid water system described above was chosen and two water molecules in the centre of the water phase, about 1.6 nm away from the surface, were removed and replaced by one Fe(II) ion, resulting in a total of 430 atoms. An MD simulation was run to equilibrate the system using the initial c-NNP. Hematite atoms and the first two interfacial layers of water were fixed in space during a short MD trajectory to equilibrate the bulk water and coordination geometry of Fe(II) before unfreezing all atoms. The same active learning procedure was carried out as explained above for aqueous Fe(II) until the network was stable (cycle in the centre of Fig. 1 main text). The c-NNP parameters used for the MD were identical to the two prior networks initially - later an extra hidden layer was added (see below). Notice that during the initial exploration of configuration space in the active learning cycle, Fe(II) remained in the bulk region, about 12-16 Å away from the surface.

Next, Fe(II) was slowly moved towards the surface using harmonic bias potentials (umbrella windows) along the distance between the ion and the oxygen surface layer in the direction of the surface normal. The surface normal is along the z-axis. A total of 14 umbrella windows were employed, with umbrella potential parameters summarized in Tab. S3. For each window active learning was performed as illustrated in Fig. 1 main text (cycle at the bottom right). The MD protocol for active learning was identical to that outlined above for aqueous Fe(II), aside from using the CSVR thermostat.[20] During active learning 312 structures of Fe(II) at distances between 1-8 Å from the interface were added to the dataset including physisorbed, monodentate and tridentate chemisorbed structures. The dataset

used to generate the final c-NNP for Fe(II) in contact with hematite(001) consisted of these structures and the merged data for aqueous Fe(II) and hematite(001)/liquid water, totalling 840 structures.

Table S1: Harmonic umbrella potentials of the form $(1/2)k(r-r_0)^2$ used for the simulation of adsorption of Fe(II) on hematite(001), where r is the distance between Fe(II) and the oxygen surface layer in the direction of the surface normal, r_0 is the equilibrium value and k the force constant.

r_0(Å)	1.30	1.55	1.85	2.05	2.20	2.75	3.00
k (kcal mol^{-1} Å^{-2})	160	100	160	240	160	40	100

r_0(Å)	3.25	3.50	4.50	5.00	5.50	6.50	7.50
k (kcal mol^{-1} Å^{-2})	50	20	20	20	20	10	10

The final c-NNP was tested against DFT reference calculations. To this end, c-NNP MD was run at each umbrella window for 500ps and 10 structures were randomly extracted for each umbrella window. After the training dataset was generated with the active learning procedure, the hyperparameters of the final c-NNP were optimised. The aim of this optimisation was to reduce the force RMSE of the committee members on the test set, without increasing computational cost drastically. The final c-NNP reports an overall force RMSE of 123.4 meV Å^{-1}, with species RMSEs displayed in Fig. S2.

The final c-NNP trained on the aqueous interface, the aqueous ion and the ion at the aqueous interface, is used to obtain the free energy profile for water dissociation from aqueous Fe(II) and for adsorption of Fe(II) on hematite(001), as detailed in the following.

Locality of 2nd generation Behler-Parrinello Neural Networks

The 2nd generation Behler-Parrinello Neural Network potential incorporates a cutoff radius to approximate the total energy of the system as a sum of atomic energies determined by each atom's local environment. In this work we use a cutoff radius of 6 Å, values very similar to this have been proven suitable for aqueous systems and interfaces.[7,23,24] Due to the dielectric screening present in liquid water, which has a dielectric constant of 78.4,[25]

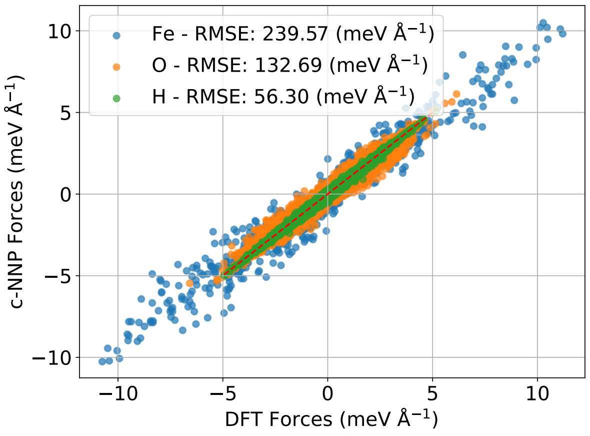

Figure S2: Test of final c-NNP trained on DFT reference data for aqueous Fe(II), hematite(001)/liquid water and aqueous Fe(II) in contact with hematite(001). The test set consists of 10 structures from each umbrella window, spanning the range of the reaction coordinate investigated and including physisorbed, monodentate and tridentate chemisorbed structures. See SI text for details.

long range electrostatics should be effectively screened, such that long-range descriptors are not required. However, there are instances where this is no longer appropriate. In these cases, a different simulation methodology would be required to incorporate the long-range electrostatic interactions and several distinct approaches have been recently suggested to do so.[26–30] These extensions, however, usually come with an increased computational cost.

In the case of the systems simulated herein, we justify the locality approximation as follows: for Fe(II) in water the RDF (Fig. 2A) between Fe-O decays to 1 at distances of 5 Å and greater, indicating no structural correlation between Fe and O beyond that distance. Note that the DFT data is almost quantitatively reproduced. This means that increasing the cutoff further wouldn't provide more information about the local environment of the atom.

In the case of the interfacial system, the primary question is if there are any long-range interactions between the interface and the ion which require long-range descriptors. To test that, we calculate the force RMSE as a function of ion-interface distance, which is displayed in Fig. S3. At distances larger than 6 Å, the ion cannot explicitly "see" the interface, while at lower distances, ion-interface interactions are explicitly included in the model. First, we

find that the ion's RMSE does not change drastically as it crosses the threshold at 6 Å. Even more importantly, the RMSE is somewhat smaller if the ion-interface interactions are not explicitly included. Both observations imply that there is no additional error introduced by omission of long-range descriptors. If long-range effects between the ion and the interface were important, we would expect the RMSE of the ion to significantly increase beyond the 6 Å cutoff, since the ML model would neglect all long-range effects, while the reference DFT calculations would include them. Therefore, it is appropriate to use a short-ranged ML methodology to reliably reproduce the underlying DFT potential energy surface.

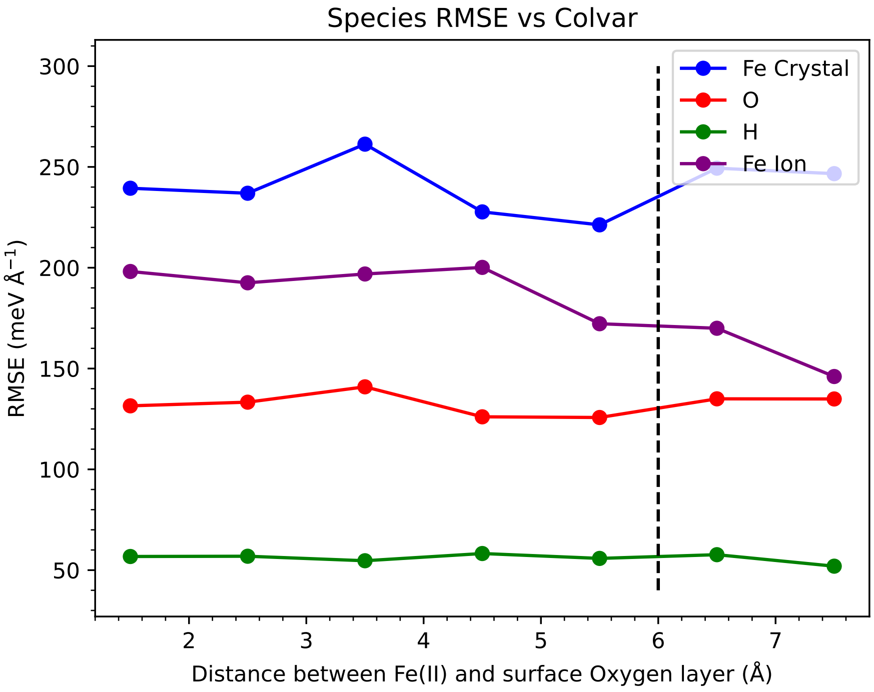

Figure S3: This plot shows the RMSE of each species from the Fe(II) ion adsorption test set resolved by the distance of the ion from the interface. Each configuration in the test set is assigned into a bin ranging from 1-8 Å, in increments of 1 Å. Next, the RMSE of the forces is calculated for each bin and then the RMSE of the forces is plotted for each species within the bin. The vertical dashed line indicates the 6 Å cutoff radius of the c-NNP model.

Free energy profile for dissociation of a first shell water molecule from aqueous Fe(II)

As outlined in the main text, in order to simulate dissociation of a first shell water molecule from Fe(II), the first step of the water exchange reaction, we chose as reaction coordinate the coordination number of the Fe(II) ion with respect to all oxygen atoms in the cell. This reaction coordinate was defined in `cp2k` with the keyword `COORDINATION` in the `COLVAR` section of `SUBSYS`. The functional form of the reaction coordinate is given by

$$\mathrm{CN} = \sum_{j}^{N_{\mathrm{O}}} \frac{1 - \left(\frac{r_{\mathrm{Fe},j}}{R_0}\right)^{NN}}{1 - \left(\frac{r_{\mathrm{Fe},j}}{R_0}\right)^{ND}}, \tag{S1}$$

where $r_{\mathrm{Fe},j}$ is the distance between the iron atom and oxygen atom j, R_0 is the distance cutoff, N_{O} is the number oxygen atoms in the supercell and the exponents NN and ND control the steepness of the step function. These were assigned values of $R_0 = 3$ Å, NN $=$ 20 and ND $=$ 40. The free energy profile was obtained by umbrella sampling in the forward direction from coordination number 6 to 5 and in the reverse direction from coordination number 5 to 6. The umbrella potentials used are summarized in Tab. S2. In each window the system was equilibrated for 10 ps using c-NNP MD and a CSVR thermostat[20] and the final structure used as the initial configuration for the next umbrella window. The final structure from each equilibration run in a given window was taken as the initial structure for the production runs of 100 ps of c-NNP MD for each window. This in turn yielded the distributions displayed in Fig. S4 for each umbrella window along the trajectory. Unbiasing of the umbrella sampling distributions in Fig. S4 was performed using the weighted histogram analysis method (WHAM) to yield the free energy profile shown in Fig. 2(C) main text.[31–33] The free energy barriers and corresponding reaction rates are summarized in Tab. 1, calculated with the reactive flux formalism.[34,35] As outlined in the main text, the frequency prefactor and transmission coefficient were estimated from a swarm of 16384 trajectories

initiated at the transition state. The normalized reactive flux was calculated ensuring that once a given trajectory reached within a standard deviation of the product minima, it was counted as a successful trajectory and the time series of the reaction coordinate was set to that value for the remainder of the trajectory. This is in line with Chandler's description of the reactive flux formalism, which is modelling the rate for one transition of reactant to product.[34] Unrestrained MD was run to calculate the mean and standard deviation of the reaction coordinate in the stable regions of the free energy profile. However, the rapid rate of the 5→6 transition didn't allow for suitable time scales to calculate these reliably. Instead, the standard deviation of the 6 coordinate well was taken. Using these definitions, the rate calculated for the 5→6 predicts the reaction should occur in about 2ps, which was in line with the observed time for our unrestrained MD in the 5-fold well before the 6-fold species formed. The impact of allowing for multiple transitions is displayed in Fig. S5, which shows the normalized reactive flux correlation function not reaching a plateau value. The transmission coefficient is calculated by averaging the final 50fs of the reactive flux correlation function.

Table S2: Harmonic umbrella potentials of the form $(1/2)k(\mathrm{CN}\text{-}\mathrm{CN}_0)^2$ used for the simulation of ligand dissociation of aqueous Fe(II), where CN, defined in Eq. S1, is the Fe(II) coordination number with respect to all oxygen atoms in the simulation cell, CN_0 is the equilibrium value and k the force constant.

CN_0	6.00	5.95	5.90	5.85	5.80	5.75	5.70
k (kcal mol^{-1})	200.0	200.0	300.0	400.0	500.0	500.0	550.0

CN_0	5.65	5.60	5.55	5.50	5.45	5.40	5.35
k (kcal mol^{-1})	550.0	600.0	600.0	600.0	600.0	600.0	600.0

CN_0	5.30	5.25	5.20	5.15	5.10	5.05	5.00
k (kcal mol^{-1})	550.0	500.0	450.0	400.0	300.0	200.0	200.0

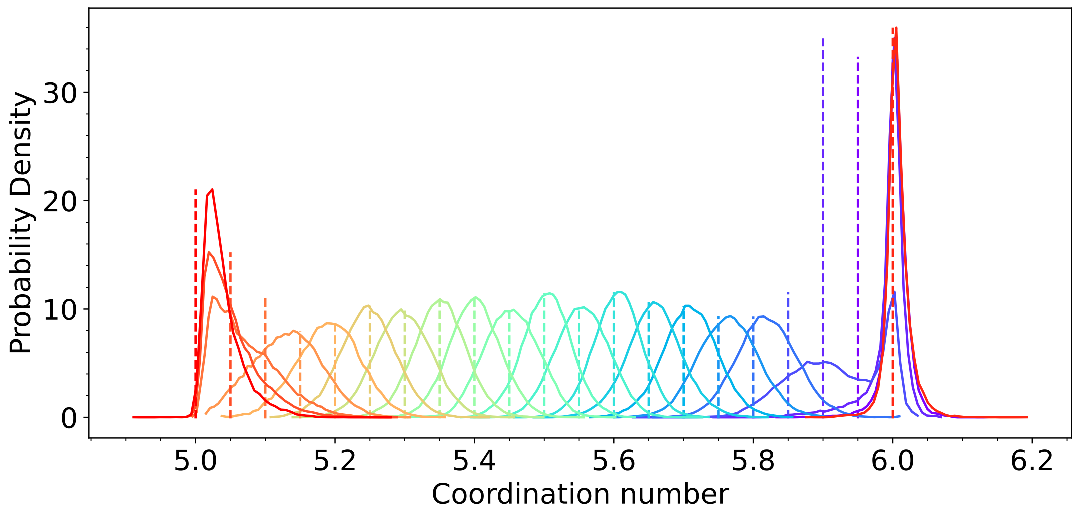

Figure S4: Umbrella sampling for the water ligand dissociation from aqueous Fe(II). Distributions of reaction coordinate values (coordination numbers) are shown for the forward sweep from coordination number 6 to 5, and very similar distributions are obtained for the backwards sweep. The equilibrium value of coordination number of each harmonic umbrella potential is shown in vertical dashed lines (see Tab. S2 for numerical values). The free energy profile obtained from unbiasing the distributions is shown in Fig. 2(C) main text. All simulations were carried our with the final c-NNP trained on the aqueous interface, the aqueous ion and the ion at the aqueous interface.

Free energy profile for adsorption of aqueous Fe(II) on hematite(001)

We again used umbrella sampling to calculate the free energy profile for adsorption of aqueous Fe(II) on hematite(001). The reaction coordinate, defined as the distance along z-axis of Fe(II) to the mean interfacial oxygen position (with the z-axis being the normal vector of the interfacial plane), and the umbrella windows (Tab. S3) were the same as previously used when the ion was moved towards the surface for the generation of the final merged c-NNP. In each window the system was equilibrated for 5 ps using c-NNP MD and a CSVR thermostat,[20] starting from an initial configuration sampled from the stable c-NNP MD trajectories generated during the active learning procedure. Recall that the system was allowed to explore the equilibrium configurations until the network was stable both in variance and force RMSE before the next window was sampled. This approach ensures that the reaction coordinate was sampled reversibly across its range. The final structure from each equili-

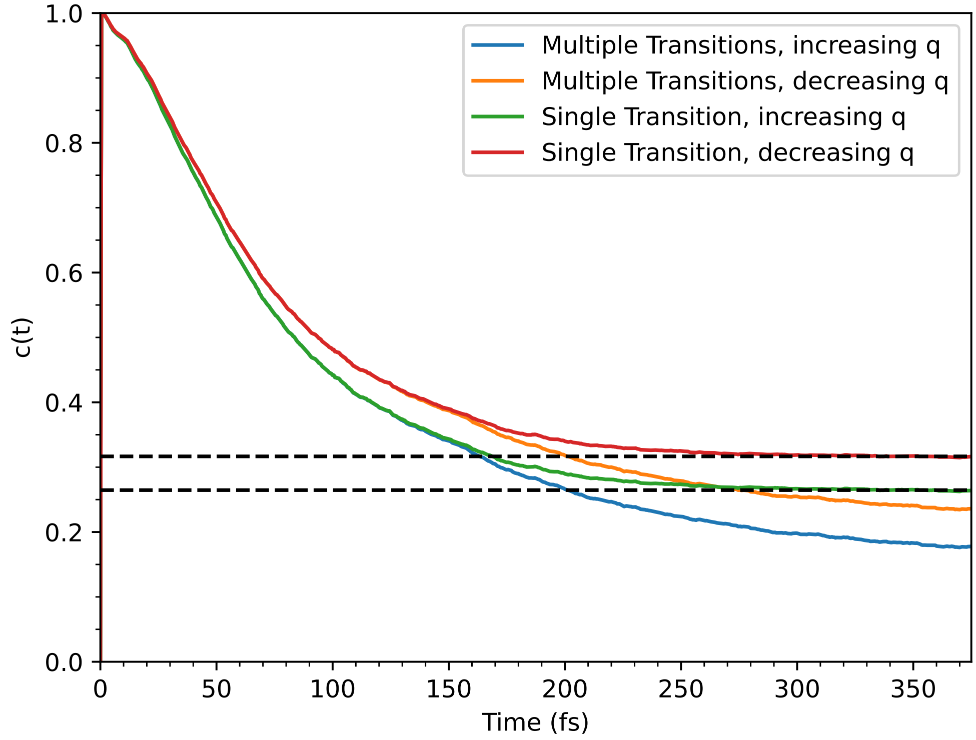

Figure S5: Reactive flux correlation functions, Eq. 9, for the water ligand dissociation from aqueous Fe(II), with and without enforcing a single reaction. When recrossings are allowed, the reactive flux correlation function does not plateau as expected for a single transition. Once single transitions are enforced, the transmission coefficient is calculated by averaging the final 50fs of the reactive flux correlation function, which does plateau on the molecular timescale.

bration run in a given window was taken as the initial structure for the production runs of 500ps of c-NNP MD for each window. The fluctuations of the reaction coordinate along the production runs is shown in Fig. S6(A) and the corresponding probability distributions in Fig. S6(C). The variance in the energy prediction across the committee members along the trajectory is shown in Fig. S6(B) for each umbrella window. The variance is reasonably flat and at approximately the same value in each umbrella window reiterating the confidence of our network in predicting energy and forces of configurations across the whole range of structures along the reaction coordinate. Unbiasing of the umbrella sampling distributions was performed using the weighted histogram analysis method (WHAM) to yield the free

energy profile shown in Fig. 3(A) main text.[31–33] Note that the free energy profile displayed in the main text shows the range of 1-6 Å only to ease comparison with the free energy profile obtained in a previous classical MD study.[36] The free energy profile was tested for convergence with respect to the simulation length in each window. The results are shown in Fig. S7. We observe that the free energy profile is converged with respect to simulation time after about 400 ps per window as there are virtually no changes in the profile when simulation time is increased from 400 ps to 500 ps. The free energy barriers and corresponding reaction rates are summarized in Tab. 1, calculated with the reactive flux formalism.[34,35] The same protocol for calculating the transmission coefficient was used as for the water dissociation reaction. Note that for trajectories whose c-NNP energy variance spikes are excluded from the analysis. The reactive flux correlation function plots for the adsorption are shown in figure Fig. S8.

Table S3: Harmonic umbrella potentials of the form $(1/2)k(r - r_0)^2$ used for the simulation of adsorption of Fe(II) on hematite(001), where r is the distance between Fe(II) and the oxygen surface layer in the direction of the surface normal, r_0 is the equilibrium value and k the force constant.

r_0(Å)	1.30	1.55	1.85	2.05	2.20	2.75	3.00
k (kcal mol^{-1} Å^{-2})	160	100	160	240	160	40	100

r_0(Å)	3.25	3.50	4.50	5.00	5.50	6.50	7.50
k (kcal mol^{-1} Å^{-2})	50	20	20	20	20	10	10

DFT MD simulation for aqueous Fe(II)

The initial geometry for the DFT MD simulation was the final frame of the FF MD simulation. The system was equilibrated for approximately 2.5ps and then a 5ps production run was performed. The 5ps of production data was used to calculate the results shown in Fig. 2(A,B) in the main text. DFT MD simulations were carried out with a time step of 0.5 fs applying a chain of Nose-Hoover thermostats,[18,19] with the aforementioned DFT setup employed.

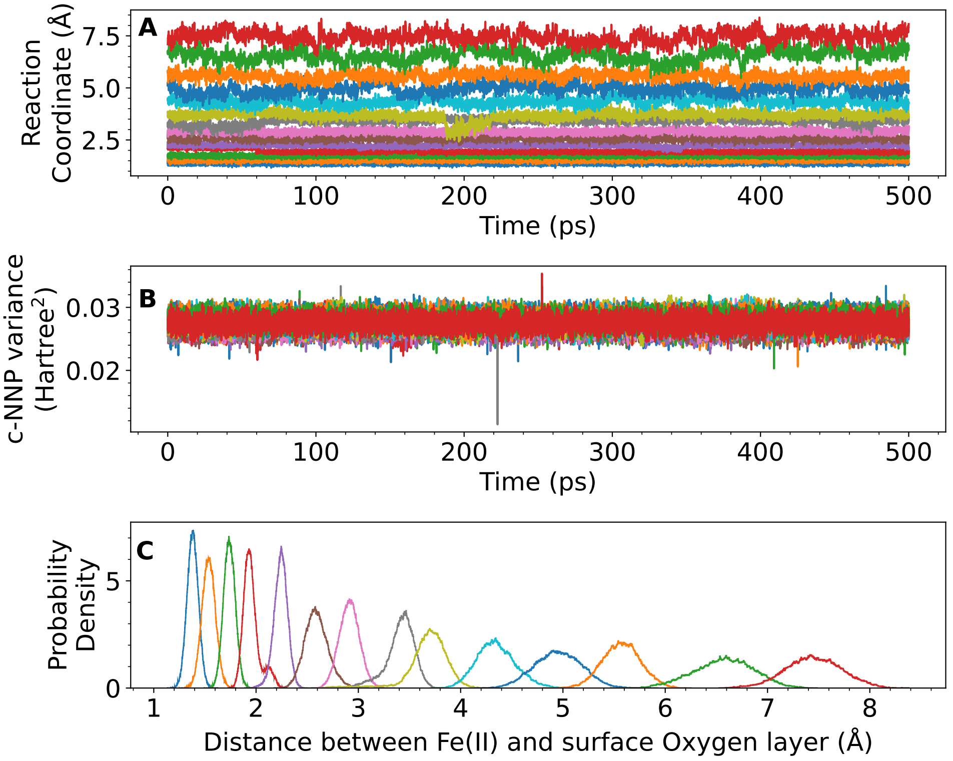

Figure S6: Umbrella sampling of adsorption of aqueous Fe(II) on hematite(001) in water. The fluctuations of the reaction coordinate (distance between Fe(II) and the oxygen surface layer in the direction normal to the surface, as defined in the x-axis label of panel (c)) is shown for all umbrella windows in (a) and the corresponding distributions are shown in (c). The umbrella potentials are defined in Tab. S3. The variance of the c-NNP energy prediction is shown for each umbrella window vs time in panel (b). The free energy profile obtained from unbiasing the distributions in (b) is shown in Fig. 3(A) main text. All simulations were carried out with the final c-NNP trained on the aqueous interface, the aqueous ion and the ion at the aqueous interface.

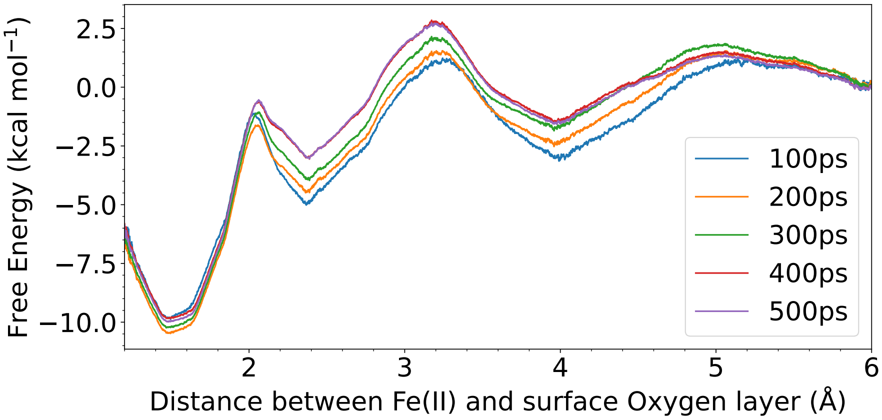

Figure S7: Convergence of free energy profile for adsorption of Fe(II) on hematite(001) in water with respect to the simulations time per umbrella window. We observe that the free energy profile converges after about 400 ps.

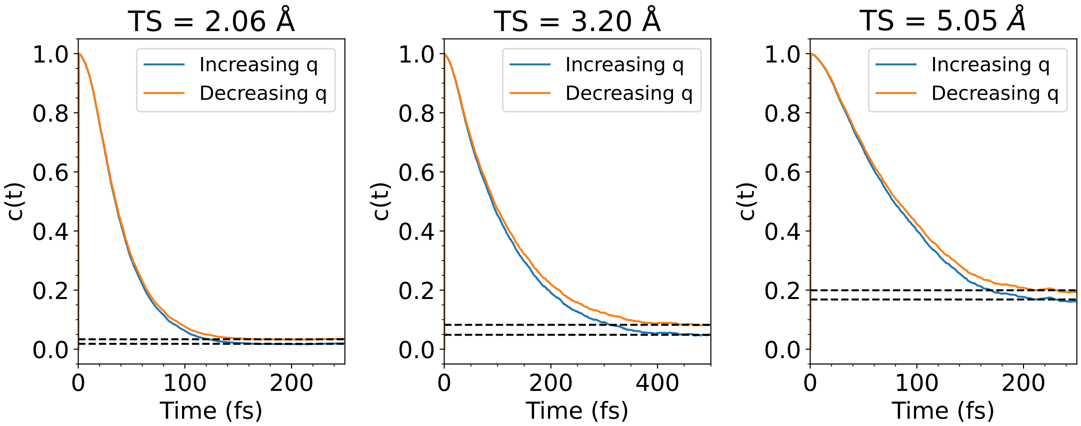

Figure S8: Reactive flux correlation functions, Eq. 9, displayed for each transition state in the adsorption of Fe(II) on hematite(001). The transmission coefficient is calculated by averaging the final 50fs of the reactive flux correlation function. Single transitions are enforced to calculate the transmission coefficient, as previously described in the text. Note that q, the reaction coordinate, is given by the distance between Fe(II) and the surface oxygen layer.

Force field-based MD simulation for aqueous Fe(II)

The initial geometry was taken from generated via the discretized grid method as described above, followed by a 20ps equilibration to 298 K. We employ the TIP3P-FB 12-6-4 potential, recently parameterized for the divalent ions.[15] The parameters for the TIP3P-FB water

model and 12-6-4 potential are given in Ref.[15,37] The charge on the Fe(II) is 2+ in this model and Lorentz Berthelot combining rules are utilised.[15] Rigid water molecules were employed using a G3X3 constraint in cp2k.[1] Classical MD simulations were carried out with a time step of 0.5 fs applying a chain of Nose-Hoover thermostats with target temperature 298 K.[18,19] For generating data for Fig. 2(A,B) in the main text, a 1ns production run was performed.

Structure and bond distances of Fe(II) adsorbed on hematite(001)

To gather unbiased statistics, structures were extracted from the corresponding minima of the free energy profile and 15 ps long free c-NNP MD simulations were performed without umbrella potentials. From these simulations, average bond distances between Fe(II) and O atoms of first shell ligands were calculated and compared to previously reported results from force field MD simulations.[36] The results are summarized in Tab. S4. Moreover, for the tridentate complex a surface density 2d histogram was computed, to check if the coordination environment on the hematite surface was the same as that from a previous classical MD study.[36] This plot is displayed in Fig. S9 and agrees with that identified previously.

Table S4: Structure and average bond distances of Fe(II) adsorbed on hematite(001).

Species	d^a (Å)		Fe-O_w^b (Å)		Fe-O_s^c (Å)	
	c-NNP[d]	FF[e]	c-NNP[d]	FF[e]	c-NNP[d]	FF[e]
Non-adsorbed	n/a	n/a	2.17	2.08	n/a	n/a
Physisorbed	4.00	4.0	2.17	2.08	n/a	n/a
Monodentate	2.40	2.5	2.16	2.07	2.21	2.42
Tridentate	1.56	1.3	2.11	2.08	2.16	2.23

[a] Distance between Fe(II) and surface layer of oxygen atoms of hematite(001) at the minimum on the free energy profile for the respective species.
[b] Average bond lengths between Fe(II) and O atoms of first shell water molecules.
[c] Average bond lengths between Fe(II) and surface O atoms of hematite(001).
[d] c-NNP MD, this work.
[e] Force field MD, average of the available model data, from Ref.[36]

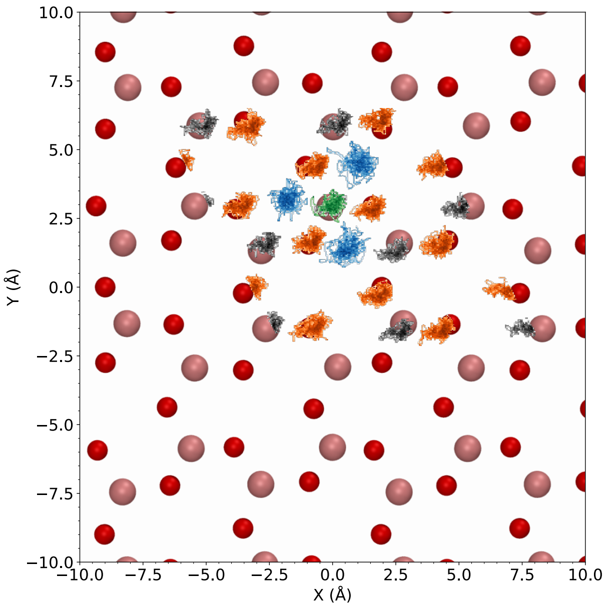

Figure S9: Surface density 2D histogram plot from the unrestrained tridentate chemisorbed complex MD projected onto a static image of the layer of surface atoms. For the histograms, surface oxygens are in orange, the surface irons are in grey, the aqueous ion is in green and the coordinated waters are in blue. For the static image, the surface oxygens are in red and the surface iron atoms in pink. This plot identifies the adsorption site as being above a surface iron, with the 3 coordinating waters staggered relative to the 3 coordinating surface oxygens. This is the same motif as identified in a previous classical MD study.[36]

References

(1) Kühne, T. D.; Iannuzzi, M.; Del Ben, M.; Rybkin, V. V.; Seewald, P.; Stein, F.; Laino, T.; Khaliullin, R. Z.; Schütt, O.; Schiffmann, F. et al. CP2K: An electronic structure and molecular dynamics software package - Quickstep: Efficient and accurate electronic structure calculations. *J. Chem. Phys.* **2020**, *152*, 194103.

(2) VandeVondele, J.; Krack, M.; Mohamed, F.; Parrinello, M.; Chassaing, T.; Hutter, J. Quickstep: Fast and accurate density functional calculations using a mixed Gaussian and plane waves approach. *Comput. Phys. Commun.* **2005**, *167*, 103–128.

(3) Krukau, A. V.; Vydrov, O. A.; Izmaylov, A. F.; Scuseria, G. E. Influence of the exchange screening parameter on the performance of screened hybrid functionals. *J. Chem. Phys.* **2006**, *125*, 224106.

(4) Pozun, Z. D.; Henkelman, G. Hybrid density functional theory band structure engineering in hematite. *J. Chem. Phys.* **2011**, *134*, 224706.

(5) von Rudorff, G. F.; Jakobsen, R.; Rosso, K. M.; Blumberger, J. Fast interconversion of hydrogen bonding at the hematite (001)–liquid water interface. *J. Phys. Chem. Lett.* **2016**, *7*, 1155–1160.

(6) Grimme, S.; Antony, J.; Ehrlich, S.; Krieg, H. A consistent and accurate ab initio parametrization of density functional dispersion correction (DFT-D) for the 94 elements H-Pu. *J. Chem. Phys.* **2010**, *132*, 154104.

(7) Schienbein, P.; Blumberger, J. Nanosecond solvation dynamics of the hematite/liquid water interface at hybrid DFT accuracy using committee neural network potentials. *Phys. Chem. Chem. Phys.* **2022**, *24*, 15365–15375.

(8) VandeVondele, J.; Hutter, J. Gaussian basis sets for accurate calculations on molecular systems in gas and condensed phases. *J. Chem. Phys.* **2007**, *127*, 114105.

(9) Guidon, M.; Hutter, J.; VandeVondele, J. Auxiliary density matrix methods for Hartree-Fock exchange calculations. *J. Chem. Theory Comput.* **2010**, *6*, 2348–2364.

(10) Schwegler, E.; Grossman, J. C.; Gygi, F.; Galli, G. Towards an assessment of the accuracy of density functional theory for first principles simulations of water. II. *J. Chem. Phys.* **2004**, *121*, 5400–5409.

(11) Gillan, M. J.; Alfe, D.; Michaelides, A. Perspective: How good is DFT for water? *J. Chem. Phys.* **2016**, *144*, 130901.

(12) Heyden, M.; Sun, J.; Funkner, S.; Mathias, G.; Forbert, H.; Havenith, M.; Marx, D. Dissecting the THz spectrum of liquid water from first principles via correlations in time and space. *Proc. Natl. Acad. Sci. U. S. A.* **2010**, *107*, 12068–12073.

(13) Gaiduk, A. P.; Zhang, C.; Gygi, F.; Galli, G. Structural and electronic properties of aqueous NaCl solutions from ab initio molecular dynamics simulations with hybrid density functionals. *Chem. Phys. Lett.* **2014**, *604*, 89–96.

(14) Schienbein, P.; Schwaab, G.; Forbert, H.; Havenith, M.; Marx, D. Correlations in the solute–solvent dynamics reach beyond the first hydration shell of ions. *J. Phys. Chem. Lett.* **2017**, *8*, 2373–2380.

(15) Li, Z.; Song, L. F.; Li, P.; Merz Jr, K. M. Systematic parametrization of divalent metal ions for the OPC3, OPC, TIP3P-FB, and TIP4P-FB water models. *J. Chem. Theory Comput.* **2020**, *16*, 4429–4442.

(16) Schran, C.; Brezina, K.; Marsalek, O. Committee neural network potentials control generalization errors and enable active learning. *J. Chem. Phys.* **2020**, *153*, 104105.

(17) Singraber, A.; Behler, J.; Dellago, C. Library-based LAMMPS implementation of high-dimensional neural network potentials. *J. Chem. Theory Comput.* **2019**, *15*, 1827–1840.

(18) Nosé, S. A molecular dynamics method for simulations in the canonical ensemble. *Mol. Phys.* **1984**, *52*, 255–268.

(19) Nosé, S. A unified formulation of the constant temperature molecular dynamics methods. *J. Chem. Phys.* **1984**, *81*, 511–519.

(20) Bussi, G.; Donadio, D.; Parrinello, M. Canonical sampling through velocity rescaling. *J. Chem. Phys.* **2007**, *126*, 014101.

(21) Gittus, O. R.; Von Rudorff, G. F.; Rosso, K. M.; Blumberger, J. Acidity constants of the hematite–liquid water interface from ab initio molecular dynamics. *J. Phys. Chem. Lett.* **2018**, *9*, 5574–5582.

(22) Boily, J.-F.; Chatman, S.; Rosso, K. M. Inner-Helmholtz potential development at the hematite (α-Fe2O3)(001) surface. *Geochim. Cosmochim. Acta* **2011**, *75*, 4113–4124.

(23) Eckhoff, M.; Behler, J. Insights into lithium manganese oxide–water interfaces using machine learning potentials. *J. Chem. Phys.* **2021**, *155*, 244703.

(24) Natarajan, S. K.; Behler, J. Neural network molecular dynamics simulations of solid–liquid interfaces: water at low-index copper surfaces. *Phys. Chem. Chem. Phys.* **2016**, *18*, 28704–28725.

(25) Fernández, D. P.; Goodwin, A. R. H.; Lemmon, E. W.; Levelt Sengers, J. M. H.; Williams, R. C. A Formulation for the Static Permittivity of Water and Steam at Temperatures from 238 K to 873 K at Pressures up to 1200 MPa, Including Derivatives and Debye-Hückel Coefficients. *J. Phys. Chem. Ref. Data* **1997**, *26*, 1125–1166.

(26) Ko, T. W.; Finkler, J. A.; Goedecker, S.; Behler, J. A fourth-generation high-dimensional neural network potential with accurate electrostatics including non-local charge transfer. *Nature Communications* **2021**, *12*, 398.

(27) O'Neill, N.; Shi, B. X.; Fong, K.; Michaelides, A.; Schran, C. To Pair or not to Pair? Machine-Learned Explicitly-Correlated Electronic Structure for NaCl in Water. *The Journal of Physical Chemistry Letters* **2024**, *15*, 6081–6091, PMID: 38820256.

(28) Gao, A.; Remsing, R. C. Self-consistent determination of long-range electrostatics in neural network potentials. *Nat. Commun.* **2022**, *13*, 1572.

(29) Grisafi, A.; Ceriotti, M. Incorporating long-range physics in atomic-scale machine learning. *The Journal of Chemical Physics* **2019**, *151*, 204105.

(30) Kabylda, A.; Vassilev-Galindo, V.; Chmiela, S.; Poltavsky, I.; Tkatchenko, A. Efficient interatomic descriptors for accurate machine learning force fields of extended molecules. *Nature Communications* **2023**, *14*, 3562.

(31) Kumar, S.; Rosenberg, J. M.; Bouzida, D.; Swendsen, R. H.; Kollman, P. A. The weighted histogram analysis method for free-energy calculations on biomolecules. I. The method. *J. Comput. Chem.* **1992**, *13*, 1011–1021.

(32) Roux, B. The calculation of the potential of mean force using computer simulations. *Comput. Phys. Commun.* **1995**, *91*, 275–282.

(33) Grossfield, A. WHAM: the weighted histogram analysis method. 2012.

(34) Chandler, D. Statistical mechanics of isomerization dynamics in liquids and the transition state approximation. *J. Chem. Phys.* **1978**, *68*, 2959–2970.

(35) Roux, B. Transition rate theory, spectral analysis, and reactive paths. *J. Chem. Phys.* **2022**, *156*, 134111.

(36) Kerisit, S.; Zarzycki, P.; Rosso, K. M. Computational molecular simulation of the oxidative adsorption of ferrous iron at the hematite (001)–water interface. *J. Phys. Chem. C* **2015**, *119*, 9242–9252.

(37) Wang, L.-P.; Martinez, T. J.; Pande, V. S. Building force fields: An automatic, systematic, and reproducible approach. *J. Phys. Chem. Lett.* **2014**, *5*, 1885–1891.
